# Subjective Improvement in Hearing After Treatment With a Manic Episode in an Elderly Patient

**DOI:** 10.7759/cureus.38624

**Published:** 2023-05-06

**Authors:** Alican Bulut, Kamadchisundaram Manoranjan, Justin Marley

**Affiliations:** 1 Psychiatry, Essex Partnership University NHS Foundation Trust (EPUT), Colchester, GBR

**Keywords:** sensorineural hearing loss, manic episodes, depression, hearing, antidepressants in elderly

## Abstract

This is a report of an 81-year-old woman presenting with hearing improvement after a reduction of antidepressant medication to manage a manic episode. The patient reported a subjective improvement in her hearing ability, not confirmed by audiometric testing. It was reported to us that she stopped using her hearing aids subsequently. This case highlights the potential for medications to impact hearing and the importance of monitoring for side effects in elderly patients with mood disorders.

## Introduction

Hearing loss is a common issue in the elderly population and is often attributed to age-related changes and exposure to noise. However, the impact of medications on hearing has been increasingly recognized as a contributing factor. Hearing impairment is one of the most common disabilities in older adults. According to a recent report by the WHO, more than 1.5 billion people live with hearing loss [1]. Hearing loss is mainly categorized as conductive, sensorineural, and mixed. The most common hearing loss in the older adult population is sensorineural hearing loss (SNHL). In the International Classification of Diseases, Eleventh Revision (ICD-11), this is described as bilateral high-frequency hearing loss associated with difficulty in speech discrimination and central auditory processing of information [2].

In this case report, we present an 81-year-old woman with a history of manic episodes, who experienced a subjective improvement in her hearing ability after the reduction of her antidepressant medication. This case sheds light on the importance of monitoring for potential side effects in elderly patients receiving psychotropic medication and highlights the need for further investigation of the relationship between mood disorders, psychotropic medications, and hearing function.

## Case presentation

An 81-year-old woman with a 26-year history of bipolar disorder presented to the community mental health team with symptoms of mania, including pressure of speech, elated mood, and lack of sleep. The manic episode lasted for two weeks at the time of the first assessment and was found to be precipitated by antidepressant medication. We did not find any evidence of delusions at the assessment. The past medical history included bipolar affective disorder, hypothyroidism, and osteoarthritis of the hips. The patient also had a 10-year history of bilateral SNHL. The medications were Lamotrigine, Mirtazapine, Olanzapine, Levothyroxine, Omeprazole, Zopiclone, and Fluoxetine. After the first assessment, Fluoxetine was stopped. Two weeks after the initial assessment, the Mirtazapine dose was gradually reduced from 45mg to 15mg in two weeks. After the reduction of Mirtazapine, the patient experienced a significant improvement in her hearing and stopped using her hearing aids during daily visits. At the follow-up assessments, she was able to communicate with the staff member without using her hearing aids. An audiology test was completed two weeks after the subjective improvement in her hearing and the result showed that her hearing has been the same for the last year.

## Discussion

In one cohort study, all classes of antidepressants were found to increase the risk of SNHL. Increased numbers of different classes of antidepressants were associated with a high risk of SNHL [3]. In another review, the authors suggest a framework, that decreased auditory input to the brain may cause the brain to compensate and facilitate speech discrimination using cross-modal plasticity [4].

Auditory perception is facilitated by the neural encoding of sound together with the transmission and processing of that information in the cortical and sub-cortical regions [5]. In this review, differences in speech discrimination in older adults with similar pure-tone audiometry results were explained by temporal deficits in the brainstem and midbrain [5].

We propose several hypotheses that may explain our patient's improvement in hearing. These are grouped into two: There was a physiological impairment that was not detected by the audiometry and there was no physiological impairment.

There was a physiological impairment not detected by audiometry

Audiometry involves pure tone tests but can also include other tests such as speech discrimination. We did not have further information on the tests that were performed but instead received a summary of the outcome from the patient on which we have based our considerations. If the audiometry involved speech discrimination then the possibilities below should be considered.

Firstly, the effects of antidepressant treatment are likely mediated in part by changes in neural plasticity [6].

The second hypothesis is that depression and mania influence hearing in this case via concentration. This in turn may improve speech discrimination even with the presence of partial hearing loss.

Thirdly, there could be an iatrogenic effect. In our patient, altered serotonergic activity in the brain may change the activity in the auditory cortex or associated areas. In one study, the authors found that increased plasma serotonin levels were found to be a biomarker of sudden sensorineural hearing loss (SSNHL) [7]. Therefore in this case there might be a link between serotonin levels and auditory processing.

Although the audiometry test result did not show any difference from previous years, the patient reported significant improvement in her hearing level. These perceived changes resulted in her stopping using hearing aids, which she used for a long period of time.

There was no physiological change

There remains the possibility that the perception of improved hearing occurred without a physiological change. In this case, the perception may represent a reverse somatoform disorder whereby there is a perception of recovered function. This may also be a delusion occurring in the context of mania where there is an overestimation of abilities. The study period and available information were not sufficient to exclude these possibilities.

As hearing impairment is a significant disability in the older adult population, this area needs further investigation. Although there are gaps in the information in our case, there are sufficient questions from the research literature to offer a framework for further considerations in this area.

## Conclusions

Hearing loss is very common in the older adult population and this can impact on quality of life. Hearing impairment is a significant problem globally and psychiatrists need to be aware of this when treating psychiatric problems. One of the drawbacks of the study was that we did not use the Naranjo algorithm. Psychosomatic causes of hearing loss in the older adult population are an area that would benefit from further research. Further research is needed into the role, if any of serotonin in hearing.
